# Machine Learning-Based Risk Stratification for Sudden Cardiac Death Using Clinical and Device-Derived Data

**DOI:** 10.3390/s26010086

**Published:** 2025-12-22

**Authors:** Hana Ivandic, Branimir Pervan, Mislav Puljevic, Vedran Velagic, Alan Jovic

**Affiliations:** 1 University of Zagreb Faculty of Electrical Engineering and Computing, Unska 3, HR-10000 Zagreb, Croatia; hana.ivandic@fer.unizg.hr (H.I.); branimir.pervan@fer.unizg.hr (B.P.); 2 University Hospital Centre Zagreb, Ulica Mije Kišpatića 12, HR-10000 Zagreb, Croatia; mislav.puljevic@kbc-zagreb.hr (M.P.); vedran.velagic@kbc-zagreb.hr (V.V.); 3 University of Zagreb School of Medicine, Šalata 2, HR-10000 Zagreb, Croatia

**Keywords:** machine learning, SHAP analysis, sudden cardiac death, implantable cardioverter defibrillator

## Abstract

Sudden cardiac death (SCD) remains a major clinical challenge, with implantable cardioverter-defibrillators (ICDs) serving as the primary preventive intervention. Current patient selection guidelines rely on limited and imperfect risk markers. This study explores the potential of machine learning (ML) models to improve SCD risk prediction using tabular clinical data that include features derived from medical sensing devices such as electrocardiograms (ECGs) and ICDs. Several ML models, including tree-based models, Naive Bayes (NB), logistic regression (LR), and voting classifiers (VC), were trained on demographic, clinical, laboratory, and device-derived variables from patients who underwent ICD implantation at a Croatian tertiary center. The target variable was the activation of the ICD device (appropriate or inappropriate/missed), serving as a surrogate for high-risk SCD detection. Models were optimized for the F2-score to prioritize high-risk patient detection, and interpretability was achieved with post hoc SHAP value analysis, which confirmed known and revealed additional potential SCD predictors. The random forest (RF) model achieved the highest F2-score (F2-score 0.74, AUC-ROC 0.73), demonstrating a recall of 97.30% and meeting the primary objective of high true positive detection, while the VC classifier achieved the highest overall discrimination (F2-score 0.71, AUC-ROC 0.76). The predictive performance of multiple ML models, particularly the high recall they achieved, demonstrates the promising potential of ML to refine ICD patient selection.

## 1. Introduction

Sudden cardiac death (SCD) is an unexpected death caused by a very fast and dangerous heart rhythm that stops the heart from pumping blood. Most often, this rhythm starts in the ventricles (ventricular tachycardia or ventricular fibrillation) and can lead to collapse and death within minutes if an electrical shock is not delivered in time [1]. Because such a condition usually happens suddenly and often in patients who could otherwise be perceived as stable, SCD is a major clinical problem. It remains a leading cause of cardiovascular mortality, particularly among individuals with left ventricular dysfunction. To address and reduce this risk, contemporary cardiology mostly uses implantable cardioverter–defibrillators (ICDs). ICDs are relatively small, implantable devices that continuously monitor the heart rhythm and can deliver an internal electric shock when a life-threatening arrhythmia appears. Large clinical trials have shown that ICDs can improve survival in patients after myocardial infarction or with heart failure and reduced left ventricular ejection fraction (LVEF), thus becoming a standard for SCD prevention and protection of high-risk patients, as established by landmark trials such as MADIT, MUSTT, and SCD-HeFT [2,3,4]. Current European guidelines also support ICD therapy in selected patients at increased arrhythmic risk [1].

To determine candidacy for ICD-based prevention therapy, contemporary clinical practice, following prevailing guideline recommendations, is largely based on LVEF. LVEF is the fraction of blood ejected from the left ventricle during systole relative to the volume of blood present at the end of diastole. Defined in such a manner, LVEF is expressed as a percentage and represents the proportion of blood pumped out of the left ventricle with each heartbeat [5]. In accordance with established guidelines, individuals with an LVEF ≤ 35% are typically considered for primary-prevention ICD implantation. Despite its widespread use, LVEF-based selection for ICD therapy remains imperfect. A substantial proportion of patients who receive an ICD solely due to reduced LVEF never experience malignant ventricular arrhythmias requiring device intervention, and conversely, clinically significant arrhythmic events may still occur in patients with only moderately reduced LVEF [6,7]. Furthermore, the benefits of ICD prevention therapy are less consistent in non-ischemic cardiomyopathy [4,8,9], which is further confirmed by Akel and Lafferty in their meta-analysis [10]. These observations highlight that reliance on a single metric, especially LVEF, is insufficient to accurately characterize individual risk of SCD. Current guidelines acknowledge these limitations and emphasize the need for more individualized risk stratification [1]. In this context, our previous CRO-INSIGHT study analyzed 614 consecutive patients with ICD or CRT-D devices implanted at a Croatian tertiary center. One of the findings was that approximately one-third of patients received appropriate ICD therapy, which implies that the majority of patients never triggered device activation, thus undergoing implantation, along with inherent procedural and long-term risks, without a clear survival benefit [11].

Considering the points discussed and the availability of large-scale routinely obtained tabular clinical data, the application of Artificial Intelligence (AI) and more specifically, Machine Learning (ML), emerges as a particularly promising avenue, especially given the recent substantial breakthroughs achieved in the field. A growing body of research has explored the application of ML to risk stratification and prediction of SCD. Systematic reviews and surveys highlight the usage of a variety of models, ranging from neural networks, support vector machines, extreme gradient boosting, to random forests, and diverse data approaches [12,13,14].

ECG-based approaches have emerged as a promising direction for SCD identification [15], with some models demonstrating the ability to predict SCD risk up to one year before the event, with performance improving as ECG recordings approach the time of SCD [16]. Multimodal ML approaches that integrate ECG and clinical data show particularly strong performance, achieving, in recent studies, AUC-ROC values up to 0.86 [17] and 0.90 [18]. Multimodality can be realized not only at the data level but also at the model level, with some studies incorporating neural networks as components of more complex model architectures for SCD detection [19]. Some approaches relied on SCD prediction based on heart rate variabilities and heartprint indices [20]. Another work has focused on reducing device burden, successfully predicting inappropriate ICD therapies (AUC-ROC 0.87) [21]. On a wider scale, models such as RF-SLAM have also accurately forecast all-cause mortality and hospitalization in ICD populations (AUC-ROC > 0.80) [22]. Beyond general ICD therapy prediction, ML has also been applied in more specialized contexts, including distinguishing sustained from self-terminating ventricular tachycardia [23], improving risk stratification in Brugada syndrome [24], or differentiating cardiac death modes in heart failure [25]. Related research has also investigated wearable-based systems for the real-time identification of early cardiac events, including heart attack and SCD, leveraging continuous ECG acquisition and embedded machine-learning models [26,27].

From the medical and clinical point of view, using AI for more precise risk stratification opens up a plethora of possibilities to spare patients from unnecessary procedures and the complications that follow them, including but not limited to lead failure, infection, inappropriate shocks, and the psychological toll of carrying a device [10,22]. At the same time, individuals at higher risk (e.g., younger patients and patients with a clearly defined arrhythmic substrate) may be identified with greater certainty and protected more effectively [4,23]. Recent studies also suggest that ML-based approaches could predict additional clinically relevant outcomes, including inappropriate therapies, hospitalizations, and mortality [21,28]. This implies that such models could eventually support, in addition to implantation decisions, long-term follow-up and therapeutic planning. All of the aforementioned aspects become even more relevant when placed within the current ESC guidelines, which acknowledged the shortcomings of an LVEF-based approach [1]. More recent EHRA documents addressed the role of digital health and AI, highlighting their potential while emphasizing the necessity for external validation and transparent clinical implementation prior to widespread practical adoption [29]. Together, these viewpoints reinforce the stance that AI is gaining recognition as a future component of SCD prevention strategies, although practical routes for its integration are still evolving.

Although multimodal approaches show strong performance, the primary aim of this study was to demonstrate that routinely collected clinical data alone can contribute to risk stratification for ICD implantation. Using retrospective data from a Croatian tertiary hospital, we applied ML models to predict appropriate ICD therapy, i.e., interventions that are likely to prevent SCD. Our ultimate goal is to move toward precision risk stratification, ensuring high-risk patients receive lifesaving therapy while minimizing unnecessary procedures in low-risk individuals. This paper, therefore, presents the performance of ML models in identifying appropriate device activation, a potentially significant step toward more refined and personalized ICD decision-making.

The rest of the paper is structured as follows. First, in Section 2, we describe the dataset used in this study, both in terms of its acquisition and preprocessing. Furthermore, we also describe the applied ML methods. In Section 3, we present the results, and in Section 4, we discuss their clinical significance. Finally, we conclude the paper in Section 5.

## 2. Materials and Methods

For this study, we used a dataset of patients who received an ICD or CRT-D device at University Hospital Centre Zagreb (hrv. KBC Zagreb) between 2009 and 2018, collected and described by Puljević et al. [11]. The dataset consisted of demographic information, device indication, and clinical parameters, including medications for 614 patients. It also contained device-derived data from the ECG and ICD systems, including information about both appropriate and inappropriate device activations. In this study, we aimed to train AI models to classify patients at greater risk of SCD, as reflected by appropriate device activations. In the first subsection, we give all the details about how the dataset was adjusted to be eligible for different models. In the second subsection, we concentrate on the methodology used in the study.

### 2.1. Dataset Adjustment

All used features with their respective short descriptions are listed in Table 1, Table 2 and Table 3. For improved readability, the features were organized into three tables according to their value type. Their original values were encoded as numerical ones to ensure compatibility with all AI models used in this study. Some features from the original dataset were omitted due to high rates of missing values, irrelevance, or details about device activation. The target feature was defined such that appropriate activations were labeled as positive instances, while both non-activations and inappropriate activations were treated as negative instances.

Patients’ IDs were assigned randomly to ensure data anonymity. The feature months was adjusted to indicate the period during which the patient had the device. Specifically, this duration represented the time from implantation until a heart transplantation, death from diverse causes, or the defined closing date of the dataset collection. Instances where a patient was tracked for less than a month were omitted, leaving the dataset with 607 instances instead of the original 614.

While the feature months potentially introduced unequal follow-up bias into the dataset, it was considered important for clinical interpretation and thus retained. Its inclusion was crucial for understanding the full spectrum of patients’ exposure to the device, enabling us to differentiate between patients with a short follow-up time who did not experience an ICD activation and those who were followed for years without any such event. It is important to note that patient follow-up continued even after the device activation until the final data collection date. Furthermore, it should be emphasized that the length of the follow-up period reflects the time until any recorded outcome (transplantation, death from different causes, or study closure), and does not imply death due to SCD. The study was cross-sectional, as the dataset was collected retrospectively. The distributions of the features used in the study were presented in [30].

The original dataset also contained a number of missing values. For the feature company, a new category (3) was used to fill the missing values. We opted for a new category to save potentially important information and to avoid introducing an additional bias to a certain ICD manufacturing company. For nyha, vt sustained, vt freq, and diuretic medication, missing values were filled with 0 based on the clinical assumption that an undocumented status implies the absence of the condition. Missing values of the vt sustained and vt freq were expected, as some patients did not suffer a ventricular tachycardia. Although these values, alongside diuretic use, had as many as 38.26% and 21.02% missing values, respectively, and present a key limitation of the study introducing imputation bias, the features were kept given their critical clinical relevance and potential informational value.

### 2.2. Experiments Description

In this study, we tested several machine learning algorithms to predict the appropriate device activation. The following models were considered: tree-based methods (decision tree—DT, random forest—RF, and extreme gradient boosting—XGBoost), Naive Bayes (both Gaussian—GNB and Multinomial—MNB), and logistic regression (LR). A soft voting classifier (VC) was also implemented and tested, as it allows combining several different types of models. Each model ran the prediction independently. The predicted probabilities were aggregated, and the final prediction of the VC for a given sample was determined by taking the class with the highest summed probability (argmax).

The models were also tested on scaled data. For the MNB model, the features were normalized, while for all other models, the features were standardized. Apart from scaling, due to a high class imbalance with only 27.51% of positive instances, data resampling was used. Both undersampling and oversampling were tested. In the case of undersampling, only the randomly selected samples of the majority class were used for training. On the contrary, for the oversampling approach, we used the SMOTE (Synthetic Minority Oversampling TEchnique) algorithm, which generates synthetic instances of the minority class based on its nearest neighbors [31].

The steps were organized in a pipeline to automate the workflow and avoid data leakage. The dataset was divided randomly into a train and test set in an 80:20 ratio. Then, a grid search with 10-fold cross-validation was performed on the training set to find the optimal model parameters. This way, the parameters achieving the highest average score across 10 folds were selected as optimal. The tested parameters for different models and their optimal values are presented in Appendix A. As the primary goal of the study was to identify patients with appropriate device activations and minimize the number of false negative instances, the models were trained to optimize the F2-score, emphasizing recall while still considering precision:

(1) $$F2\text{-}\mathrm{Score}=\frac{5\cdot Precision\cdot Recall}{4\cdot Precision+Recall}$$

To further improve the performance, we also tested training the models on the AUC-ROC score with classification threshold adjustment. After the model had been trained, the optimal classification threshold for F2-score maximization was determined using 10-fold cross-validation. For each model, accuracy, precision, recall, F1-score, F2-score, and AUC-ROC score were reported on the test set. Confusion matrices for all trained models are available in Appendix B.

The tests were divided into two groups. In the first group, all described features were used for model training, while in the second, a filter method was applied to select the most relevant features. Highly correlated features were removed, and only the top 10 features ranked by ANOVA F-value were retained. The feature correlation matrix is presented in Appendix C.

**Figure 1:**
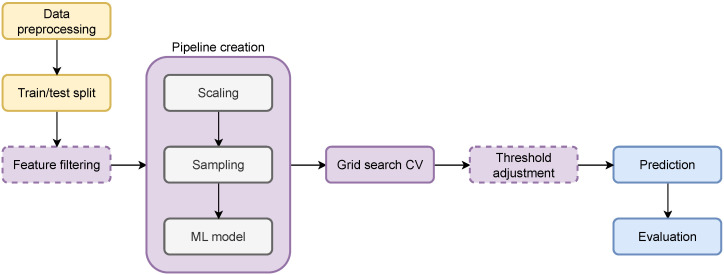
Workflow diagram.

The overall workflow is presented in Figure 1. The yellow blocks were applied to the whole dataset, the purple ones were applied to the train set, and the blue ones to the test set. Data preprocessing included all steps described in Section 2.1 (value encoding, feature selection, feature adjustment, outlier removal, value imputation). The dashed blocks mark optional steps (feature filtering and threshold adjustment) depending on different scenarios.

Shapley additive explanations (SHAP) values were calculated to explain how the models reached their conclusions. The method presented by Lundberg and Lee [32] assigns an importance value to each feature for each particular prediction. By doing so, it aims to reveal the features that had the highest impact on the overall output of the model. The method was applied to all models except the VC, as it consists of several different models. The parameters for each model in the VC were optimized individually; thus, the feature importance of each constituent model remained the same as when used independently.

Finally, although it would be preferable to evaluate our workflow on additional datasets to further strengthen the robustness of our findings, this was not attainable. The primary limitation lies in the substantial heterogeneity of available datasets. A valid external comparison would require a dataset containing the same feature set as ours, which is not feasible given that datasets used in related work differ considerably, often incorporating non-tabular data such as imaging data or physiological signals.

## 3. Results

The results are organized in two subsections depending on the features used for appropriate ICD activation prediction. The first subsection presents the results obtained from training the models on the entire available set of features, while the second subsection reports the models’ performance after training on the top 10 selected features.

### 3.1. All Features

In the first case scenario, all 32 features (except the patients’ IDs) described in Section 2.1 were used. Table 4 presents the results after training the models on the F2-score. In our previous study [30], we reported the performance of the LR model chosen due to its simplicity and interpretability. None of the other tested models managed to outperform its F2-score of 0.73 or F1-score of 0.60, but the VC consisting of LR, pruned DT, and RF achieved a higher AUC-ROC score. Nevertheless, its accuracy and precision were lower, while the recall remained unchanged. The Multinomial NB achieved the highest accuracy and precision, but the lowest recall, resulting in the lowest F2-score. In the case of NB and LR, scaling and sampling of the dataset improved the models’ performance, whereas in the pruned DT and VC, it did not lead to performance gains.

Results obtained after the models had been trained on AUC-ROC, and the classification threshold was shifted to maximize the F2-score, are presented in Table 5. This approach enhanced the models’ ability to recognize patients who suffered an ICD activation (true positive—TP cases), but it did not result in an improvement in precision. Recall and F2-score increased for all models, while all models except the DT suffered a decrease in accuracy and precision. The LR model was able to recognize as many as 97.3% of the patients from the test set who suffered an ICD activation. Although the model was able to recognize almost all positive instances, accuracy and precision were significantly lower when compared to the LR model trained directly on the F2-score. The best performance was achieved through the LR model after oversampling the training dataset, while the performance of VC (which consisted of 5 models: LR, kNN, Multinomial NB, pruned DT, and Gaussian NB) improved after scaling the data. Although the LR model achieved the highest F2-score, the Multinomial NB model achieved the highest AUC-ROC score of 0.73. Despite achieving the highest F1-score, the DT classifier showed the lowest recall among all evaluated models. This supports the use of the F2-score as a more appropriate metric for this task, as it prioritizes recall to a greater extent.

**Figure 2:**
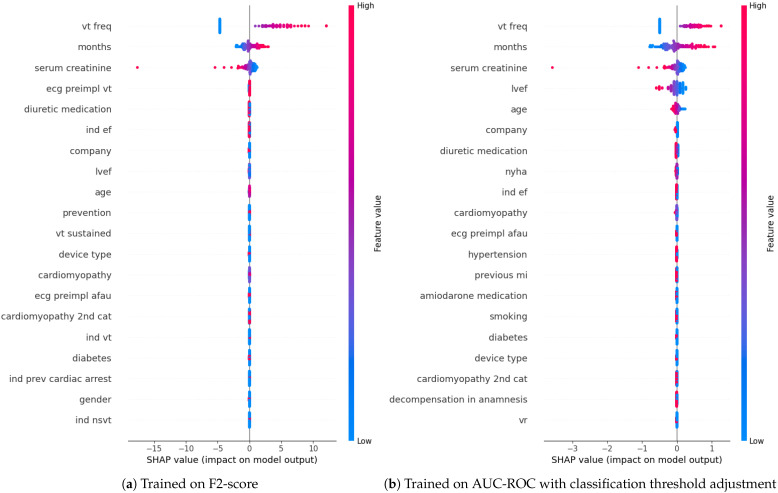
SHAP values for LR as the best-performing model trained on all features for both training approaches.

Regarding feature importance analyzed with SHAP values, all models identified VT frequency as the most important factor, with higher values increasing the likelihood of an appropriate ICD activation, and thus indicating a higher risk of SCD. For both the LR and Multinomial NB models, (1) longer periods of patient follow-up and (2) lower serum creatinine levels had the same impact. In the second case, apart from the mentioned features, the LR model tended to predict SCD in patients with (1) lower LVEF values and of (2) younger age, while the Gaussian NB leaned toward the same prediction for cases with (1) secondary prevention, (2) presence of VT in ECG as an indication for ICD implantation, (3) sustained VT, and the absence of (4) low ejection fraction and (5) diuretic medication. Although the pruned DT trained on the F2-score only considered the VT frequency, the DT trained on the AUC-ROC score also assigned high importance to (1) the patient follow-up duration and (2) decompensation in anamnesis. In this model, longer follow-up and a history of decompensation increased the chance of predicting a higher risk of SCD. The SHAP values for the LR model trained on the F2-score and on the AUC-ROC score are presented in Figure 2.

### 3.2. Filtered Features

In this scenario, we trained the models only on ten (10) selected features. The only feature removed due to high correlation with other features was vt freq, which showed a strong correlation with ecg preimpl vt. An alternative configuration, where the ecg preimpl vt feature was excluded, and vt freq was retained, was also evaluated, but it yielded inferior results. This result was expected, given that the vt freq feature was missing whenever ecg preimpl vt equals 0, corresponding to cases where no VT event occurred. The features with the highest ANOVA F-value, which were used in this case, were (1) device type, (2) ind prev cardiac arrest, (3) ind ef, (4) ind vt, (5) prevention, (6) ecg preimpl vt, (7) vt sustained, (8) ecg preimpl vf, (9) diuretic medication, and (10) months.

As in the previous case, the results are organized into two tables. Table 6 presents the results for the four model types trained on filtered data to maximize the F2-score. This time, the Gaussian NB outperformed the Multinomial one, but its overall performance was comparable. It achieved the highest accuracy and precision, but the lowest recall, with an F2-score of less than 0.63. XGBoost also achieved higher accuracy and precision, but lower recall and F2-score compared to the scenario of using all features. In this case, the best-performing model was the VC, which consisted of a Gaussian NB, LR, and a pruned DT. All models performed better on either an undersampled or oversampled dataset, while the performances of XGBoost and LR improved after scaling the dataset.

Model training on AUC-ROC score with a classification threshold adjustment for F2-score maximization was also applied to filtered features, with results shown in Table 7. In this case, all models performed better on a scaled dataset, achieving a recall above 0.89. RF model stood out as the best-performing one, reaching a recall of 0.97 and an F2-score of 0.74. While Gaussian NB reached a similar F2-score, it did so with higher accuracy and precision, but a lower recall. The performance of all models, except the VC, remained the same regardless of the sampling technique. VC, which used voting of a pruned DT, RF, and a kNN model, performed better after using undersampling. Overall, training on the AUC-ROC score increased the recall of all models, but also decreased their accuracy and precision.

**Figure 3:**
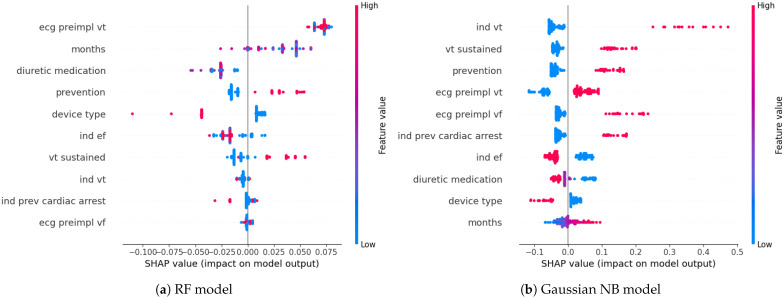
SHAP values for the best-performing models trained on the filtered features and AUC-ROC score.

In the previous case, where all 32 features were available, VT frequency was highlighted by all models as the most relevant feature. In the filtered features scenario, however, VT frequency was excluded due to high correlation with other features. Nevertheless, SHAP analysis revealed that (1) preimplantation ventricular tachycardia, (2) secondary prevention, and (3) a longer follow-up period increased the predicted risk of SCD across all models. Moreover, (1) ICD as the implanted device type, (2) sustained VT, and (3) the absence of diuretic medication had the same impact in all models except the LR. Furosemide and Spironolactone decreased the models’ outputs more than other diuretic medications, reducing the chance of ICD activation even further. Another impactful factor for Gaussian NB in both cases (when trained on F2-score and AUC-ROC score) and RF and LR trained on AUC-ROC score was the low ejection fraction as an indication for ICD implantation. The models’ outputs increased when a low ejection fraction wasn’t used as an indicator. Additional high-impact factors for the Gaussian NB trained on the AUC-ROC score were (1) preimplantation ventricular fibrillation in the ECG and (2) prior cardiac arrest as an indication for ICD implementation. The SHAP values for the best-performing models in this scenario are presented in Figure 3. In this case, the two best-performing models were both trained on the AUC-ROC score.

## 4. Discussion

The main aim of this study was not to deprive any patient of an ICD device but to explore the possibilities of utilizing ML for enhancing the prescreening of patients considered for ICD implantation. Relying on the valid ESC recommendations at the time, all individuals in our cohort received ICD devices. Nevertheless, the majority of implanted patients never receive device therapy and thus undergo an invasive procedure with associated procedural risks, potential long-term side effects, and psychological burden without a tangible survival benefit. Appropriate activation occurred in only 27.5% of patients in our cohort. These statistical results raised suspicion that additional factors beyond LVEF might increase the risk of SCD.

In our previous work [30], we tested an LR model trained on the F2-score, achieving an accuracy of 0.65, precision of 0.46, recall of 0.86, F2-score of 0.73, and AUC-ROC score of 0.74. In the same study, we analyzed the features using the coefficients learned during the model training. In this study, our goal was to test different models by optimizing them for the F2-score directly or the AUC-ROC score with classification threshold adjustment. We selected the F2-score as the primary performance metric to keep the patients’ well-being a priority. Derived from the definition given in Equation (1) in Section 2.2, F2-score places greater weight on recall, a metric which describes the rate of correctly identified positives in a subset of all positives (i.e., true positives and false negatives), which aligns with our goal of correctly identifying as many true positives as possible, even at the expense of a higher false positive rate since all patients currently receive an ICD device. This approach minimizes the risk of missing a high-risk patient while allowing us to explore the added value of an ML-based stratification.

In the study, we leveraged several ML models to predict SCD using an appropriate ICD activation as a surrogate indicator. Tree-based methods (DT, RF, and XGBoost), Naive Bayes (GNB and MNB), logistic regression, and voting classifier were trained to differentiate between patients with and without appropriate ICD activation. The experiments were divided into two scenarios depending on the used features. While in the first one, we used all available clinical, device-related, and demographic data, in the second one, we filtered the top 10 features with the highest ANOVA F-values. In both cases, we compared the models’ performance when trained directly on the F2-score and when trained on the AUC-ROC score with classification threshold adjustment for the F2-score optimization.

**Figure 4:**
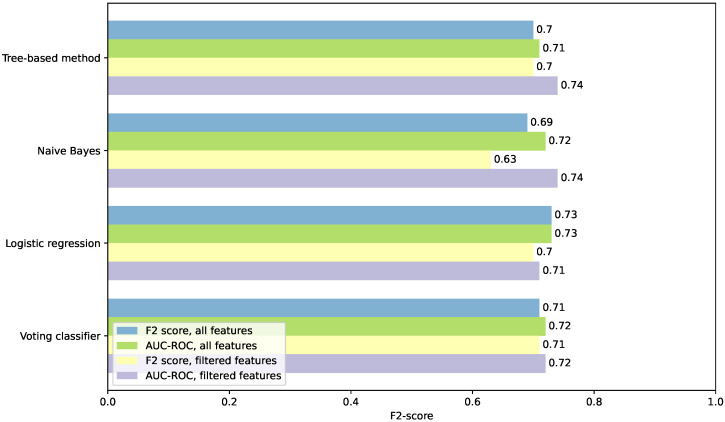
F2-scores comparison for all the tested scenarios and models.

Figure 4 sums up the F2-scores of all models and scenarios. As seen in the graph, training the models on the AUC-ROC score and later threshold adjustment for an optimal F2-score improved the F2-score of all models in both scenarios, except for the LR model trained on all features, when it remained consistent. Feature filtering had a positive impact on the tree-based and NB models in the second scenario, with models achieving an F2-score of 0.74.

Although our previous study’s LR model achieved an F2-score of 0.73, it correctly predicted 86.5% of positive instances [30]. Now, the best-performing model, RF trained on the AUC-ROC score and filtered features, achieved a similar F2-score of 0.74, but managed to recognize as many as 97.3% positives, leaving only a single instance as a false negative. Although this model achieved lower accuracy and precision, maximizing recall was our primary goal. On average, the best recall was achieved by models trained on filtered features and the AUC-ROC score with classification threshold adjustment.

**Figure 5:**
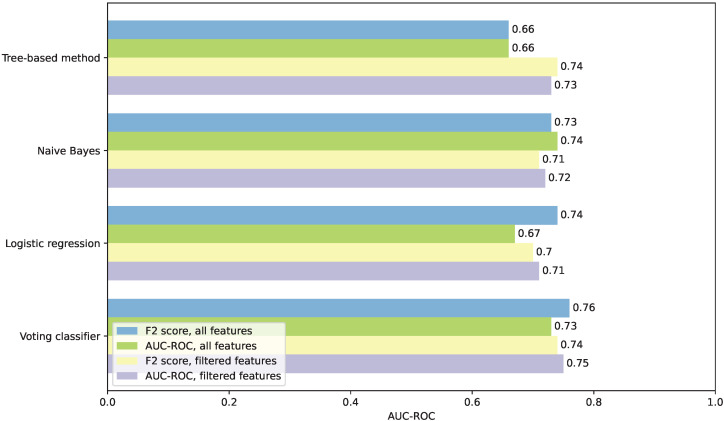
AUC-ROC scores comparison for all the tested scenarios and models.

We also opted to display and discuss the AUC-ROC score, as it poses a metric that we found to be the most commonly reported in literature, thus allowing for a simpler and fairer comparison. The highest AUC-ROC score we achieved was 0.76, reached by a soft voting classifier based on an LR, pruned DT, and RF, trained on all features and the F2-score. The AUC-ROC scores for all of the tested models are summed up for clarity in Figure 5. On average, the VC models achieved the highest AUC-ROC score. Training the model directly on the AUC-ROC score and later classification threshold adjustment improved the final AUC-ROC score of the NB classifier trained both on all and filtered features, and the LR model and the VC model trained only on filtered features.

Placed within a broader context, our findings indicate that the predictive performance achieved in this study is on par with results reported in the existing literature. Our highest AUC-ROC of 0.76 using the soft voting classifier matches the median performance reported in relevant surveys, namely Nazar et al. [12] (0.76), Kolk et al. [13] (0.79), and Barker et al. [14] (0.80). It is important to note that feature sets used by many studies included in these surveys differ substantially, often incorporating richer data such as imaging, ECG recordings, and other multimodal clinical information, which may positively influence prediction. Furthermore, this heterogeneity limits the possibility of a direct, objective performance comparison. To address this, we selected a subset of studies that are more comparable to our work in terms of dataset composition, as summarized in Table 8. In this context, our findings are on par with Nakajima et al. [25]. While Yu et al. [33] report a higher AUC-ROC of 0.89, the study has not undergone peer review, and Tateishi et al. [21] report an AUC-ROC of 0.869, whereas our model achieves a marginally higher F1 score (0.561 vs. 0.533). On the other hand, Deng et al. [34] report only a C-index, thus rendering the direct comparison infeasible. Nonetheless, our results demonstrate clear potential for risk stratification of SCD based solely on routinely collected tabular clinical data, highlighting the meaningful contribution of our work to the current body of knowledge.

In our previous paper [30], we analyzed the feature importance of the LR model using its coefficients. (1) Ventricular tachycardia, particularly its (2) higher frequency and (3) sustained episodes, along with a (4) longer follow-up period and (5) secondary prevention, emerged as factors that increased the likelihood that the LR model would predict an appropriate ICD activation. On the other hand, (1) EF as the indication for ICD implantation and (2) diuretic medication, especially furosemide and spironolactone, had the opposite impact. The model was also less likely to predict SCD for female patients and patients with CRT-D devices.

Now, we refined the feature analysis using SHAP values, ensuring a uniform, modelagnostic approach to interpretability. This approach allowed clinicians to understand how the models reach their conclusions, rather than perceiving them as a black box. The SHAP analysis validated the initial conclusions drawn from the LR model and demonstrated comparable feature importance patterns across multiple models, especially those trained on filtered features.

While feature importance was less consistent when all features were included, high VT frequency consistently emerged as the most important feature across all models. For LR and NB models, (1) longer follow-up and (2) low serum creatinine levels were also considered important independently of training the models on F2 or AUC-ROC scores. When training on the AUC-ROC score, additional features emerged as important: (1) lower LVEF and (2) younger age for LR, (1) secondary prevention, (2) VT in ECG as an indication for ICD implantation, (3) sustained VT, (4) the absence of low ejection fraction, and (5) the absence of diuretic medication for the NB model, and (1) longer follow-up period and (2) decompensation in anamnesis for pruned DT. All these factors enhanced the probability that the models would predict an SCD.

As mentioned, the feature importance was more consistent for models trained only on a filtered subset of 10 features, although VT frequency was excluded due to its high correlation with other features. All models recognized (1) preimplantation VT, (2) secondary prevention, and (3) a longer follow-up period as important factors increasing the risk of SCD prediction. All models except the LR also highlighted the influence of (3) ICD as the implanted device type, (4) sustained VT, and (5) the absence of diuretic medication on SCD prediction. Again, furosemide and spironolactone had a positive impact on the outcome, reducing the chances that the models would predict SCD. In all models trained on the AUC-ROC score, the absence of LVEF as an indication for ICD implantation increased the chances of SCD. (1) Preimplantation ventricular fibrillation and (2) prior cardiac arrest as an indication for ICD implementation had the same impact, but of higher importance only for the Gaussian NB trained on the AUC-ROC score.

The consistency of these results indicates that there might be additional factors impacting SCD risk. The SHAP analysis revealed that patients with high-frequency sustained VT and patients with a prior history of cardiac arrest were more prone to SCD. The analysis identified diuretic medications as influential to the models’ predictions, with patients receiving diuretics, especially furosemide and spironolactone, being less likely to be classified as positive.

However, caution is needed when interpreting these results as the association between medication use and outcomes is not necessarily causal. Diuretic therapy is closely intertwined with disease severity, existing comorbidities, and the overall treatment approach. The features identified are likely reflections of complex and interrelated clinical characteristics rather than independent effects. Consequently, the observed positive effect of the diuretic therapy may be a proxy for underlying patient characteristics or management intensity, rather than the diuretics demonstrating a direct positive effect. Given the retrospective and observational nature of the study, these mechanisms cannot be disentangled within the current modeling approach. At the same time, this observation aligns with prior evidence demonstrating the survival benefit of mineralocorticoid receptor antagonists in advanced heart failure, providing a plausible clinical context for the model-identified association without implying causality.

Similarly, the observation that the patients with CRT-D were less likely to experience appropriate ICD activation may reflect differences in patient selection, disease characteristics, or treatment approach, rather than a direct stabilizing effect of the device itself. It is crucial to emphasize that these observations were derived solely from the interpretation of the models’ reasoning, and that only through rigorous, extensive, and long-term clinical validation could any potential clinical relevance or benefit be responsibly considered. Nevertheless, our results confirm the potential of ML as a complement to existing frameworks, facilitating the refinement of risk stratification towards more individualized decision-making, as already suggested by several studies [24,25,35].

Several limitations of the study must be acknowledged, particularly regarding the models’ performance. While demonstrating high sensitivity (recognizing 97.3% positive instances in the best case), the models exhibited a relatively low precision (37.9% for the best overall model), translating to a significant number of false positives.

This outcome was influenced by several factors. Firstly, the prediction task by itself was highly challenging, as the objective was to identify factors influencing SCD beyond the scope of the current ESC guidelines. Secondly, the models were deliberately trained to maximize recall as a fundamental clinical priority. Given the critical nature of missing a true positive patient, the models were optimized for F2-score, which places twice the weight on recall compared to precision. Although several models achieved higher accuracy and precision (up to 66.4% and 46.8%, respectively), it was at the cost of drops in recall. The strategy of optimizing the models for F2-score successfully minimized false negatives, but resulted in a decrease in precision. Thirdly, the dataset itself presented several constraints: (1) it was restricted solely to patients who received an ICD implant according to current ESC guidelines, (2) it was inherently imbalanced (only 27.51% of positive instances), (3) the features were not entirely independent, (4) some feature missing values were imputed to retain the potentially relevant features, and (5) there was a substantial variability in the patients’ follow-up time. The latter two constraints may have introduced bias into the analysis.

These findings highlight the potential of applying ML to SCD risk stratification. However, to fully realize this potential and to progress towards clinical practice, the models’ precision must be improved. Achieving this will likely require more complex models that can incorporate different data modalities, such as ECG signals or Cardiac magnetic resonance (CMR) images, which could boost the performance. Once the precision is enhanced, the models could serve as a valuable assistant to cardiologists, specifically for risk refinement among patients already selected for ICD implantation according to the current ESC guidelines. Nonetheless, it is crucial to note that because the current ESC guidelines classify all included patients as positive, every true negative prediction classified by the model represents an improvement over the existing risk stratification principle.

## 5. Conclusions

In this paper, we applied several ML models to predict which patients would experience an SCD event, thus undergo an ICD activation using clinical, device-related, and demographic data from patients who underwent ICD or CRT-D device implantation at the University Hospital Centre Zagreb.

The models were trained to maximize F2-score, thereby prioritizing recall and identification of true positives. The models were optimized directly on the F2-score or by adjusting the classification threshold after training on the AUC-ROC score. The highest overall AUC-ROC (0.76) was obtained with a VC combining LR, a pruned DT, and RF trained on F2-score and all features. Additionally, RF trained on the top 10 features ranked by ANOVA F-value and AUC-ROC score achieved the highest F2-score (0.74), correctly predicting 97.3% of appropriate ICD activations, thus meeting the study’s primary objective of maximizing the identification of true positives. Model interpretation confirmed known SCD risk factors (high-frequency VT, reduced LVEF) and identified additional predictors such as longer follow-up period, low serum creatinine, younger age, secondary prevention, sustained VT, ICD as the implanted device, and the absence of diuretics.

High recall rates demonstrated the promising potential of ML for SCD stratification, but exhibited relatively low precision, which should be improved before introducing the models into clinical practice. Nonetheless, they already demonstrate a valuable enhancement of the existing ESC guidelines by enabling better risk stratification within the patients selected for device implantation. Future efforts should prioritize validation in larger cohorts and incorporate new data modalities to boost precision and generalizability, while maintaining the achieved high sensitivity.

## Figures and Tables

**Table 1:** Features used in the study with integer values.

Feature	Description
id	Patient’s ID number
age	Patient’s age at the time of implantation
lvef	Left ventricular ejection fraction
vt freq	Ventricular tachycardia frequency
serum creatinine	Serum creatinine level
months	The length of patient follow-up in months

**Table 2:** Features used in the study with binary values where 0 stands for no and 1 for yes.

Feature	Description
ind prev cardiac arrest	Prior cardiac arrest as an indication for ICD implantation
ind ef	Low ejection fraction as an indication for ICD implantation
ind vt	Ventricular tachycardia as an indication for ICD implantation
ind nsvt	Non-sustained ventricular tachycardia as an indication for ICD implantation
ecg preimpl vt	Preimplantation ventricular tachycardia in the ECG
ecg preimpl vf	Preimplantation ventricular fibrillation in the ECG
ecg preimpl afau	Preimplantation atrial fibrillation (AF)/atrial undulation (AU) in the ECG
ecg preimpl lbbb	Preimplantation left bundle branch block in ECG
previouos mi	History of myocardial infarction
decompensation in anamnesis	History of decompensation
diabetes	History of diabetes
hypertension	History of hypertension
thyroid disease	History of thyroid condition
smoking	Patient is a smoker
amiodarone medication	Patient is on amiodarone medication
acei/arb medication	Patient is on ACEI/ARB medication
beta-blocker medication	Patient is on beta-blocker medication

**Table 3:** Features used in the study with coded values.

Feature	Values	Description
gender	0—men, 1—women	Patient’s gender
device type	0—ICD, 1—CRT-D	Type of the implanted device
vr	0—vr, 1—dr	Single (vr) or dual (dr) chamber device
company	0–3	Device manufacturer company id
prevention	0—primary, 1—secondary	Prevention type
cardiomyopathy	0–7	Cardiomyopathy type
cardiomyopathy 2nd cat	0—non-ischemic, 1—ischemic	Cardiomyopathy type (2nd level classification)
nyha	0–4	New York Heart Association functional class
vt sustained	0—non-sustained, 1—sustained	Ventricular tachycardia duration
diuretic medication	0—no, 1—yes 2—Furosemide and Spironolactone	Patient is on diuretic medication

**Table 4:** Prediction based on all available features with models trained on F2-score.

Method	Accuracy	Precision	Recall	F1-Score	F2-Score	AUC-ROC (95% CI)
Tree-based method	0.5738	0.4051	0.8649	0.5517	0.7048	0.6560 (0.5854–0.7301)
Naive Bayes	0.6639	0.4677	0.7838	0.5859	0.6905	0.7310 (0.6076–0.7913)
Logistic regression [30]	0.6475	0.4571	0.8649	0.5981	0.7339	0.7396 (0.6351–0.8229)
Voting classifier	0.5902	0.4156	0.8649	0.5614	0.7111	0.7583 (0.6652–0.8439)

**Table 5:** Prediction based on all available features with models trained on AUC-ROC score with adjusted classification threshold.

Method	Accuracy	Precision	Recall	F1-Score	F2-Score	AUC-ROC (95% CI)
Tree-based method	0.5902	0.4156	0.8649	0.5614	0.7111	0.6639 (0.5672–0.7576)
Naive Bayes	0.5656	0.4024	0.8919	0.5546	0.7174	0.7428 (0.6446–0.8328)
Logistic regression	0.4918	0.3711	0.9730	0.5373	0.7347	0.6747 (0.5453–0.7494)
Voting classifier	0.5328	0.3864	0.9189	0.5440	0.7203	0.7294 (0.6331–0.8256)

**Table 6:** Prediction based on filtered features with models trained on F2-score.

Method	Accuracy	Precision	Recall	F1-Score	F2-Score	AUC-ROC (95% CI)
Tree-based method	0.6148	0.4306	0.8378	0.5688	0.7045	0.7397 (0.6244–0.8089)
Naive Bayes	0.6311	0.4333	0.7027	0.5361	0.6250	0.7113 (0.6160 –0.8099)
Logistic regression	0.5738	0.4051	0.8649	0.5517	0.7048	0.6881 (0.5906–0.7816)
Voting classifier	0.5902	0.4156	0.8649	0.5614	0.7111	0.7367 (0.6361–0.8269)

**Table 7:** Prediction based on filtered features with models trained on AUC-ROC score with adjusted classification threshold.

Method	Accuracy	Precision	Recall	F1-Score	F2-Score	AUC-ROC (95% CI)
Tree-based method	0.5082	0.3789	0.9730	0.5455	0.7407	0.7329 (0.6375–0.8264)
Naive Bayes	0.6148	0.4342	0.8919	0.5841	0.7366	0.7186 (0.6249–0.8092)
Logistic regression	0.5328	0.3837	0.8919	0.5366	0.7051	0.7138 (0.6186–0.8105)
Voting classifier	0.5164	0.3778	0.9189	0.5354	0.7143	0.7479 (0.6532–0.8335)

**Table 8:** State of the Art—Dataset-wise comparable studies.

Study	Dataset	Model	Results (Best)
Nakajima et al. (2022) [25]	Demographics Clinical factors Comorbidities Medications Laboratory values I-MIBG indices	LR, RF, Gradient boosted trees SVM, Naive Bayes Nearest neighbors	LR AUC-ROC 0.725
Tateishi et al. (2023) [21]	Demographics ECG parameters Clinical features Medications	Extra-trees classifier Gradient boosting Classifier CatBoost classifier Extreme gradient boosting Light gradient boosting machine	Extra-trees classifier AUC-ROC 0.869 F1 0.533
Yu et al. (2022) [33]	Demographics Lifestyle factors Clinical factors Medications Laboratory values ECG variables	RF-SLAM	AUC-ROC 0.89
Deng et al. (2022) [34]	Demographics Laboratory values Comorbidities Medications ECG findings Echocardiographic indices	EN-Cox, RSF, SSVM, XGBoost	XGBoost C-index 0.794 (*p* < 0.001)

**Figure A1:**
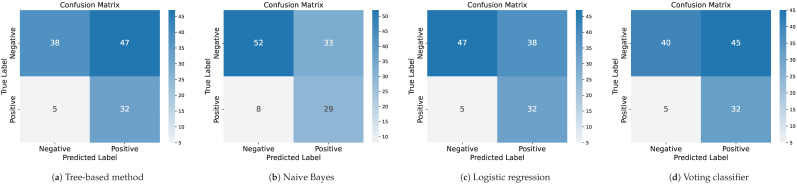
Confusion matrices for models trained on F2-score and all available features.

**Figure A2:**
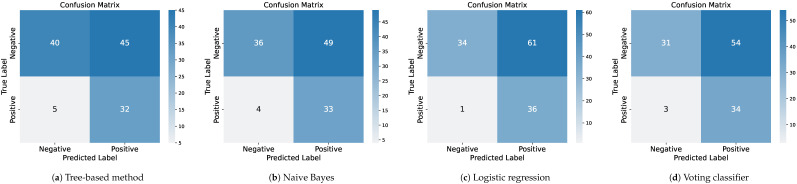
Confusion matrices for models trained on AUC-ROC score and all available features.

**Figure A3:**
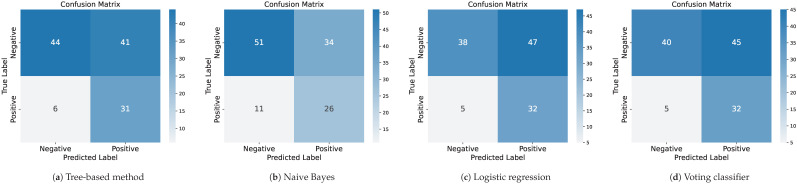
Confusion matrices for models trained on F2-score and filtered features.

**Figure A4:**
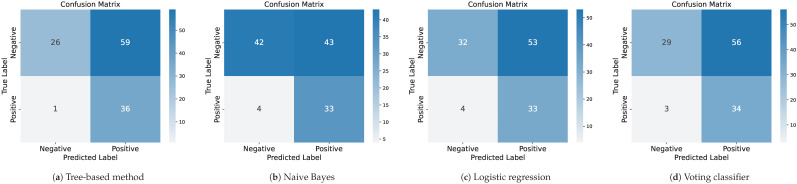
Confusion matrices for models trained on AUC-ROC score and filtered features.

**Figure A5:**
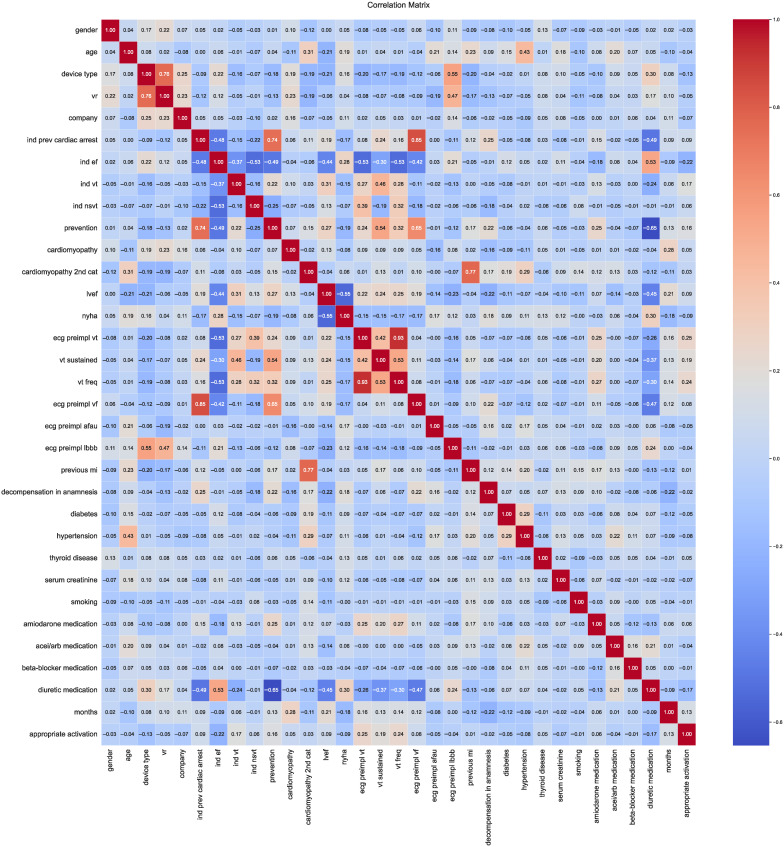
Feature correlation matrix.

## Data Availability

Inquiries regarding the dataset used in this paper should be addressed to mislav.puljevic@kbc-zagreb.hr.

## Abbreviations

The following abbreviations are used in this manuscript:

ACEI	angiotensin-converting enzyme inhibitors
AF	Atrial fibrillation
AI	Artificial intelligence
ANOVA	Analysis of Variance
ARB	Angiotensin receptor blockers
AU	Atrial undulation
AUC	Area-under-the-curve
AUC-ROC	Area under the receiver operating characteristic curve
CMR	Cardiac magnetic resonance
DT	Decision tree
ECG	Electrocardiogram
EF	Ejection fraction
ESC	European Society of Cardiology
GNB	Gaussian Naive Bayes
ICD	Implantable cardioverter-defibrillator
KNN	K-nearest neighbors
LBBB	Left bundle branch block
LR	Logistic regression
LVEF	Left ventricular ejection fraction
MI	Myocardial infarction
ML	Machine learning
MNB	Multinomial Naive Bayes
NB	Naive Bayes
NSVT	Non-sustained ventricular tachycardia
NYHA	New York Heart Association
RF	Random forest
RF-SLAM	Random Forests for Survival, Longitudinal, and Multivariate (data analysis)
ROC	Receiver operating characteristic
SCD	Sudden cardiac death
SHAP	Shapley additive explanations
TP	True positive
VC	Voting classifier
VF	Ventricular fibrillation
VT	Ventricular tachycardia
XGBoost	Extreme gradient boosting

## Appendix A. Model Parameters

The models were optimized using a 10-fold cross-validation grid search. The ranges of the tested parameters are presented in Table A1. Besides the reported models, k-nearest neighbors and support vector machine models were also tested, but the results were omitted due to insufficient performance. Nevertheless, both models were included in the grid search of the voting classifier.

**Table A1:** Tested model parameters in the 10-fold cross-validation grid search.

Model	Parameters
Decision tree	criterion = [“gini”, “entropy”, “log_loss”], max_depth = [None, 5, 10, 15, 20], min_samples_split = [2, 5, 10]
Pruned decision tree	criterion = [“gini”, “entropy”, “log_loss”], max_depth = [None, 5, 10, 15, 20], min_samples_split = [2, 5, 10] ccp_alpha = 25 alphas from the pruning path
Random forest	criterion = [“gini”, “entropy”, “log_loss”], max_depth = [None, 5, 10, 15], min_samples_split = [2, 5, 10], n_estimators = [5, 75, 100, 125, 150, 200]
XGBoost	learning_rate = [0.01, 0.05, 0.1, 0.2, 0.5], max_depth = [3, 5, 7, 9], n_estimators = [50, 100, 150, 200]
Gaussian Naive Bayes	var_smoothing = [$1\times 10^{-9}$, $1\times 10^{-8}$, $1\times 10^{-7}$, $1\times 10^{-6}$, $1\times 10^{-5}$]
Multinomial Naive Bayes	alpha = [0.1, 0.5, 1.0], fit_prior = [True, False]
Logistic regression	*C* = [0.001, 0.01, 0.1, 1, 10], penalty = [“l1”, “l2”], solver = [“liblinear”, “saga”]
K-nearest neighbors	n_neighbors = [3, 5, 7, 9, 11], weights = [“uniform”, “distance”], metric = [“euclidean”, “manhattan”, “chebyshev”]
Support vector machine	*C* = [0.1, 1, 10], kernel = [“linear”, “rbf”], gamma = [0.1, 1, “scale”]
Voting classifier	All possible subsets of 3, 6, and 9 optimized classifiers.

The following tables list the optimal parameters of all trained models. Table A2 and Table A3 define the parameters used for models trained on all features. Models in Table A2 were trained directly on F2-score, and models in Table A3 on AUC-ROC score with classification threshold adjustment to optimize the F2-score.

Table A4 and Table A5 present the optimal parameters for models trained only on filtered features. Table A4 gives the parameters of models trained on F2-score, while those in Table A5 were trained on AUC-ROC score with classification threshold adjustment.

**Table A2:** Optimal parameters for models trained on F2-score and all available features.

Model	Sampling	Scaling	Parameters
Tree-based method (pruned DT)	No	No	ccp_alpha = 0.02392, criterion = ‘gini’, max_depth = None, min_samples_split = 2
Naive Bayes (Multinomial)	SMOTE	Yes	alpha = 0.5, fit_prior = True
Logistic regression [30]	Undersampling	Yes	*C* = 0.001, penalty = ‘l2’, solver = ‘liblinear’
Voting classifier	No	No	pruned DT (ccp_alpha = 0.02392, criterion = ‘gini’, max_depth = None, min_samples_split = 2), RF (criterion = ‘entropy’, max_depth = 5, min_samples_split = 5, n_estimators = 75), LR (*C* = 0.001, penalty = ‘l2’, solver = ‘liblinear’)

**Table A3:** Optimal parameters for models trained on AUC-ROC score with adjusted classification threshold and all available features.

Model	Sampling	Scaling	Parameters
Tree-based method (DT)	No	No	criterion = ‘entropy’, max_depth = 5, min_samples_split = 10
Naive Bayes (Gaussian)	No	No	var_smoothing = $1\times 10^{-5}$
Logistic regression	SMOTE	No	*C* = 1, penalty = ‘1’, solver = ‘saga’
Voting classifier	No	Yes	pruned DT (ccp_alpha = 0.02392, criterion = ‘gini’, max_depth = None, min_samples_split = 2), GNB (var_smoothing = $1\times 10^{-9}$), MNB (alpha = 0.5, fit_prior = True) LR (*C* = 0.1, penalty = ‘l1’, solver = ‘liblinear’) kNN (metric = ‘euclidean’, n_neighbors = 3)

**Table A4:** Optimal parameters for models trained on F2-score and filtered features.

Model	Sampling	Scaling	Parameters
Tree-based method (XGBoost)	SMOTE	Yes	learning_rate = 0.01, max_depth = 3, n_estimators = 100
Naive Bayes (Gaussian)	SMOTE	No	var_smoothing = $1\times 10^{-6}$
Logistic regression	Undersampling	Yes	*C* = 0.1, penalty = ‘l1’, solver = ‘liblinear’
Voting classifier	Undersampling	No	pruned DT (ccp_alpha = 0.02302, criterion = ‘gini’, max_depth = None, min_samples_split = 2), RF (criterion = ‘entropy’, max_depth = 5, min_samples_split = 10 n_estimators = 150), LR (*C* = 0.001, penalty = ‘l2’, solver = ‘liblinear’)

**Table A5:** Optimal parameters for models trained on AUC-ROC score with adjusted classification threshold and filtered features.

Model	Sampling	Scaling	Parameters
Tree-based method (RF)	Undersampling	Yes	criterion = ‘entropy’, max_depth = 5, min_samples_split = 2, n_estimators = 200
Naive Bayes (Gaussian)	No	No	var_smoothing = $1\times 10^{-9}$
Logistic regression	No	Yes	*C* = 0.01, penalty = ‘l2’, solver = ‘saga’
Voting classifier	Undersampling	Yes	pruned DT (ccp_alpha = 0.02302, criterion = ’gini’, max_depth = None, min_samples_split = 2), RF (criterion = ‘entropy’, max_depth = 5, min_samples_split = 2, n_estimators = 5), kNN (metric = ‘manhattan’, n_neighbors = 11)

## Appendix B. Confusion Matrices

Figure A1 and Figure A2 present the confusion matrices for all models trained on all available features and F2 score in the first case, and AUC-ROC score with threshold adjustment in the second case. Additionally, Figure A3 and Figure A4 show the confusion matrices for all models trained on filtered features. While Figure A3 provides the confusion matrices of data tested on models trained on the F2 score, Figure A4 shows the confusion matrices for models trained on AUC-ROC with threshold adjustment.

As highlighted throughout the whole study, the main goal was to maximize the model performance, bearing in mind the importance of avoiding false negative predictions even at the cost of more false positive predictions. It is important to emphasize that, under the current ESC guidelines, all patients were considered to be at high risk for SCD and received an ICD implant. Therefore, from the medical point of view, all these patients were considered positive. Nevertheless, the majority of these patients never experienced a device activation, suggesting there might be additional features influencing the patient’s risk of SCD. Having this in mind, the model’s performance can be considered as a solid ground for future research. At a cost of a higher rate of false positive classifications, the models managed to recognize a high majority of patients who suffered an ICD activation, while still recognizing a considerable number of patients without appropriate device activations.

## Appendix C. Feature Correlation

Figure A5 presents the Pearson correlation matrix for all feature pairs investigated in this study. The features were considered highly correlated if the correlation coefficient exceeded 0.90. As seen in the figure, the only features that met this criterion were ecg preimpl vt and vt freq.
